# Postprandial Head-drops: Insight into Systemic Effects of Ocular Timolol Preparation in Elderly

**DOI:** 10.7759/cureus.4780

**Published:** 2019-05-30

**Authors:** Syed Hamza Bin Waqar, Aiman Rehan, Muhammad Zammam

**Affiliations:** 1 Internal Medicine, Civil Hospital Karachi, Karachi, PAK; 2 Internal Medicine, Dow University of Health Sciences, Karachi, PAK; 3 Miscellaneous, Dow University of Health Sciences, Karachi, PAK

**Keywords:** timolol, syncope, head-drop, glaucoma, side-effect, timolol, beta-blockade, syncope, systemic, head-drop

## Abstract

Timolol is a nonselective beta-adrenergic antagonist with no intrinsic sympathomimetic activity. Conditions like intraocular hypertension (IOH) and primary open angle glaucoma (POAG) warrant the use of a topical ocular preparation of timolol. It works to effectively lower the intraocular pressure (IOP) in patients with glaucoma but due to its unique pharmacodynamics, it also poses some very striking systemic side-effects. These include heart block, arrhythmias, and syncopal episodes. Herein, we present a challenging case of a previously known healthy elderly 67-year-old female, known case of POAG, who presented to the clinic with a very peculiar complaint, i.e. episodes of her head dropping down on to the table multiple times after she was done eating her food. This led to a cascade of diagnostic tests being employed encompassing cardiovascular, endocrinal, neurological, and gastrointestinal systems; all of which came out to be negative. Finally, after a very thorough literature review, it was established that timolol was the true culprit. The cessation of the drug immediately resulted in lasting relief.

## Introduction

Glaucoma has been labeled as the disease of the aging eye. Primary open angle glaucoma (POAG) is one of its variants for which timolol is often used as one of the two main first-line agents, the other being prostaglandin analogs. Although topical, the effects in a subset of elderly people have been vigorous and require special mention in literature. It is known to be a nonselective beta-adrenergic antagonist which acts by causing blockade of sympathetic firing in the nerve endings of ciliary epithelium, thereby decreasing the production of aqueous humor and lowering the raised intraocular pressure in glaucoma. Unfortunately, it can also lead to cardiopulmonary side effects. Timolol is passed in the plasma via nasal epithelium and bypasses the metabolism of the liver. Despite being present in low plasma concentration, it is deemed adequate to cause cardiovascular symptomatology ranging from palpitations to heart blocks and syncopal episodes [1]. We were presented with a very challenging case of a 67-year-old elderly female who had a known history of POAG and was now complaining of losing consciousness after eating food. After going through a myriad of diagnostic studies and ruling out all organic causes, delving deep into literature; timolol was found to be the contributing factor. These episodes halted after cessation of timolol from the regimen.

## Case presentation

A 67-year-old previously healthy female with a known diagnosis of POAG for five years presented to the clinic with a new onset of loss of consciousness which had started two weeks ago. She was doing perfectly fine when she experienced her first episode of loss of consciousness (LOC) which occurred all of a sudden causing her head to drop on to the table, followed by three more episodes before she finally presented to the clinic. She had these episodes after eating food. These syncopal events did not progress up the course of her illness. The episodes were brief, ranging from a few seconds to a few minutes. She did not experience any aura, lightheadedness, narrowing of the vision, diaphoresis, and shortness of breath, palpitation or a funny chest sensation before passing out. There were no shaky movements and she would regain complete consciousness after the episodes resolved. She denied tongue biting, frothing from the mouth, or urinary or fecal incontinence. She was started two months ago on timolol ocular preparation of 0.5% at night time daily, which she used to take 15-30 min before dinner. She denied any history of skipping meals, nausea, vomiting, alteration of bowel habits, or alcohol intake. No headache, weakness, numbness, or tingling were reported. She had a past surgical history of laparoscopic cholecystectomy that was done 10 years back. She had no significant past medical, psychiatric, or family history of a similar illness.

On admission, she was fully responsive, alert, and oriented with normal effect. The patient was afebrile with a pulse of 67 beats per minute (BPM), blood pressure (BP) of 123/67 mmHg, and respiratory rate of 13/min. Her abdomen was nondistended, nontender with normoactive bowel sounds and no organomegaly. The neurological exam for bulk, tone, power, and reflexes was insignificant for any finding. Sensations and joint position sense were intact. Cerebellar signs of co-ordination and cranial nerves two till 12 were intact and with no observed nystagmus or visible tremors. The cardiovascular exam was normal with regular rate and rhythm and no added sounds or murmurs. The pulmonary exam was normal with bilateral audible breath sounds clear to auscultation with no added wheeze. The patient had no lymphadenopathy, edema, conjunctival pallor, jaundice, rashes, or tightening of the skin.

Orthostatic vitals were also obtained in both the supine and standing position but revealed no significant difference in the blood pressure reading.

The complete blood count (CBC) showed a total leukocyte count (TLC) of 3.78 x 109/L with neutrophils being 89% and lymphocytes being 6%. Platelets were 430 x 109/L. Erythrocyte sedimentation rate and C-reactive protein levels were within normal limits. The coagulation profile was normal as well. The electrolyte panel indicated normal potassium, calcium, and sodium levels. The serum and urine osmolality was normal as well.

The glycosylated hemoglobin (HbA1c) level was 4.6% with a random blood sugar level of 89 mg/dL (normal: 79-160 mg/dL). Her blood sugar did not drop during or after the consumption of the meal. Serum insulin levels and C-peptide levels were investigated to check for the remote possibility of insulinoma but turned out to be normal as well. Ultrasound abdomen exhibited biliary duct dilatation of 7 mm, which was consistent with post-cholecystectomy status. The pancreas could not be visualized and hence endoscopic ultrasound (EUS) was done to check for any echogenic change in the pancreas but turned out to be negative. CT scan of the abdomen was also done but returned negative. A gastric emptying study was also done to look for any delays in emptying and concomitant blood sugar levels were also drafted but they were insignificant for suspected dumping syndrome.

She had head-drops after having meals which drifted our diagnosis towards more central causes as of epilepsy or a space-occupying lesion in the brain. In order to be certain, electroencephalography was done which returned to normal. MRI of the brain was also conducted but showed no growth or changes in intensity.

Finally, to exclude cardiac causes of syncope, electrocardiography, and echocardiography were done which also failed to indicate a cause. Holter monitoring was advised which turned out to be the only pertinent study showing bradyarrhythmia especially post-meal that is within 30 min of timolol maleate 0.5% drop application. Her heart rate went to 46 bpm, but she remained hemodynamically stable throughout the course with mean BP being 108/76 mmHg. Surprisingly, she did not show any palpitations.

Her regimen for POAG was then changed from timolol 0.5% to latanoprost 0.005% ophthalmic solution, a prostaglandin F2 analog, which not only aided in lowering the intraocular pressure (IOP) but also halted the syncopal head-drops that the patient was experiencing. On her six months follow up visit, the patient happily concluded that her syncopal episodes had completely resolved.

## Discussion

Timolol is a nonselective adrenergic beta-blocker with no intrinsic sympathomimetic activity. Its topical ocular preparation is considered amongst the first-line agent for lowering the IOP in patients with POAG, raised IOP secondary to traumatic cataract, and intraocular hypertension (IOH). It was first introduced in 1978 and since then has been used in ophthalmology as a wonder drug, especially in elderly patients, to help with glaucoma [1-2].

It acts at the sympathetic nerve endings by blocking the firing of neurons in the conjunctival epithelium thereby decreasing the production of aqueous humor and lowering the IOP. When applied topically, 80% of the timolol is drained via the nasolacrimal duct and the solution makes its way to the nasal mucosa where it is absorbed into the bloodstream. Its actions start a few minutes after the application, peaks four hours after, and lasts for about 24 h [3]. The absorbed stream is metabolized by a definite set of enzymes, the cytochrome P450 2D6 system in the liver. Some candidates are poor metabolizers of timolol leading to a high concentration of plasma timolol in the system. Although topical and despite being present in less than 0.1 ng/mL in plasma at its peak concentration, it is still sufficient enough to cause cardiovascular and pulmonary consequences. Its systemic effects are well documented in the literature [1-3]. A dose of one drop of 2.5%-0.5% solution to each eye is equivalent to a 5-10 mg oral dose exposing the patient to the adrenergic beta-blocking effects [4]. In addition, pharmacodynamics is also very important in the elderly. Beta receptor occupancy of timolol decreases with age and given the 12 hourly administration protocol of timolol, this leads to extensive plasma concentration and thus unwanted systemic effects. This helps to explain the systemic effects of timolol in the elderly population [5]. It causes alteration in the heart rate, diastolic blood pressure, cardiac output, exercise-tolerance, and stroke volume [4]. Its use in individuals who have cardiovascular disease or limited cerebral reserve is of special concern, as they are more prone to developing adverse effects [1].

Timolol has been reported to cause a lot of cardiovascular problems in various studies. In the case-series by Müller et al, three cases were reported which highlighted bradyarrhythmia and systolic and diastolic orthostatic hypotension as the cause of syncope in the patient [1]. Another study published by Rana et al. showed three side-effects that were concomitantly present in the patient leading to his demise. These included low blood sugar levels, sinus bradycardia with variable heart block, and orthostatic hypotension [2]. It is also known to cause sick-sinus syndrome and third-degree heart blocks [6].

In one study by Pratt et al., the relationship between bradyarrhythmia and hospitalization was observed and it showed that patients who were males and had a mean age of 82.6 were the most affected by the systemic side-effects. It also pointed out that symptomatic bradycardia increased in the 31st to 180 days after timolol was started [7].

Despite many case reports pointing towards the cardiovascular side effect profile of timolol, a meta-analysis conducted by Pinnock et al. showed that ophthalmic timolol preparation is not associated with cardiovascular mortality [8]. All such cases where timolol was the culprit in causing syncopal events, cessation of drug, and follow-up showed complete remission. It was established that in elderly patients, latanoprost and other prostaglandin analogs were a safer option as they predisposed to minimal cardiovascular side-effects as compared to beta-blockers [1-2]. Hydrogel formulation having 0.1% timolol is also considered an alternative if, for any reason, prostaglandin analogs cannot be utilized [4].

## Conclusions

Drug-induced systemic side effects should be kept on the list of differentials in all geriatric patients presenting with syncope. Timolol, in particular, should be kept in mind as this will not only provide an appropriate and timely diagnosis but will also prevent the tedious diagnostic workup that the patient would have to go through. Extensive literature should be made available to physicians working closely with such an age group and special caution should be exercised in assessing patients with glaucoma. A close liaison of physicians and ophthalmologists is also very important in such cases. Patients should be made aware of the adverse effects that the drug might cause and be advised to follow-up if they develop any of these. To conclude, prostaglandin analogs like latanoprost and effective follow-up of patients can be used in the elderly as an alternative tool to avoid the systemic havoc that one is predisposed to with timolol.
